# Single-Cell RNA Sequencing Uncovers Neutrophil Clusters Associated with Autoimmune Neuroinflammation

**DOI:** 10.21203/rs.3.rs-8012709/v1

**Published:** 2025-11-19

**Authors:** Yong Wang, William Turbitt, Lianna Zhou, Zhaoqi Yan, Sweta Patel, Wei Yang, Zhang Li, Jessica Buckley, Robert Welner, William Meador, Chander Raman, Hongwei Qin, Etty Benveniste

**Affiliations:** 1918 University Blvd, University of Alabama at Birmingham, Birmingham, AL, USA 35294; 1918 University Blvd, University of Alabama at Birmingham, Birmingham, AL, USA 35294; EMD Serono, Inc., Billerica, MA, 01821; 1918 University Blvd, University of Alabama at Birmingham, Birmingham, AL, USA 35294; 1918 University Blvd, University of Alabama at Birmingham, Birmingham, AL, USA 35294; Gladstone Institute of Neurological Disease, San Francisco, CA 94158; 1918 University Blvd, University of Alabama at Birmingham, Birmingham, AL, USA 35294; 1918 University Blvd, University of Alabama at Birmingham, Birmingham, AL, USA 35294; Weill Cornell College of Medicine, New York, NY 10021; University of Alabama at Birmingham; 1918 University Blvd, University of Alabama at Birmingham, Birmingham, AL, USA 35294; Department of Medicine, University of Alabama at Birmingham, Birmingham, AL 35294; Department of Neurology, University of Alabama at Birmingham, Birmingham, AL 35294; Department of Dermatology, University of Alabama at Birmingham, Birmingham, AL 35294; 1918 University Blvd, University of Alabama at Birmingham, Birmingham, AL, USA 35294; 701 19th Street South, ALGEN, Suite 835, University of Alabama at Birmingham, Birmingham, AL, USA 35294

**Keywords:** Suppressors Of Cytokine Signaling 3 (SOCS3), Serum Amyloid A3 (SAA3), Brain-targeted Experimental Autoimmune Encephalomyelitis (btEAE), Single-cell RNA Sequencing (scRNA-Seq), SAA3 (Serum amyloid A 3), Neutrophils

## Abstract

Multiple sclerosis (MS) is an autoimmune demyelinating disease of the central nervous system (CNS) characterized by multifocal inflammation and axonal degeneration, driven by innate and adaptive immune cells. The Janus Kinase (JAK)/Signal Transducers and Activators of Transcription (STAT)/Suppressors Of Cytokine Signaling (SOCS) pathway regulates immune cell activity, with SOCS proteins functioning as negative regulators. Using the Experimental Autoimmune Encephalomyelitis (EAE) model of MS, our prior work demonstrated that mice lacking *Socs3* in myeloid cells (*Socs3*^ΔLysM^) developed severe, brain-targeted EAE (btEAE), with increased cerebellar infiltration of activated neutrophils.

To define neutrophil-specific roles, we generated mice with *Socs3* deletion restricted to neutrophils (*Socs3*^ΔLy6G^). Following MOG-induced EAE, these mice exhibited clinical features identical to *Socs3*^ΔLysM^ mice, including severe cerebellar demyelination, increased cerebellar infiltration of activated neutrophils and CD4^+^ T-cells, and clinical symptoms of both btEAE and classical EAE (cEAE), the latter involving the spinal cord (SC). Cerebellar neutrophils from *Socs3*^ΔLy6G^ mice exhibited a primed, inflammatory phenotype with elevated reactive oxygen species, neutrophil extracellular traps (NETs) and heightened production of pro-inflammatory cytokines/chemokines. Neutrophil depletion alleviated btEAE, confirming their pathogenic role.

Single-cell RNA Sequencing (scRNA-Seq) of cerebellum (CB) and SC neutrophils revealed five clusters in naïve and EAE mice, with expansion of two clusters (Neu2 and Neu4) in *Socs3*^ΔLy6G^ mice with EAE. Neu2, Neu3 and Neu4 clusters showed high expression of *Saa3*, *Il1b* and *Cxcl2*, with Neu4 enriched in cytokine signaling pathways and inflammatory responses. Strikingly, Saa3 mRNA and protein expression were markedly increased in the CB and SC of *Socs3*^ΔLy6G^ mice with EAE compared to controls. Translationally, the human orthologue SAA1 was significantly elevated in plasma from MS patients relative to healthy controls.

Collectively, these findings demonstrate that *Socs3* deficiency unleashes pathogenic neutrophil activity in *Socs3*^ΔLy6G^ mice with EAE. They further reveal neutrophil heterogeneity within the inflamed CNS, define inflammatory transcriptional states and identify Saa3/SAA1 as potential biomarkers and therapeutic targets to attenuate autoimmune neuroinflammation.

## INTRODUCTION

Multiple Sclerosis (MS) is an autoimmune disease of the central nervous system (CNS) characterized by immune-mediated demyelination, neuroinflammation, and progressive neurodegeneration [1–4]. While adaptive immune cells such as T and B lymphocytes are well established as key drivers of MS and its animal model, Experimental Autoimmune Encephalomyelitis (EAE), the roles of innate immune cells remain less defined [3, 5, 6]. Over twenty FDA-approved disease-modifying therapies reduce relapse rates in relapsing-remitting MS; however, none halt long-term progression, underscoring the need to identify pathogenic and protective immune subsets through high-resolution profiling [7].

The Janus Kinase/Signal Transducer and Activator of Transcription (JAK/STAT) pathway, activated by more than 70 cytokines, is central to immune regulation [8–11]. Aberrant STAT activation has been reported in MS, Alzheimer’s disease (AD), and Parkinson’s disease (PD) [8, 12–16]. Suppressors Of Cytokine Signaling (SOCS) proteins serve as negative feedback regulators of JAK/STAT signaling [9, 17–20], and dysregulated *Socs3* expression in MS correlates with enhanced STAT3 activation [21, 22]. Our prior work showed that pharmacologic JAK/STAT inhibition ameliorates EAE by suppressing pathogenic T-cell differentiation, dampening myeloid activation, and limiting leukocyte infiltration into the CNS [8, 23].

EAE manifests as distinct clinical subtypes: classical EAE (cEAE), with ascending paralysis and spinal cord (SC) inflammation, and brain-targeted EAE (btEAE), marked by ataxia, tremors, and cerebellar (CB) inflammation[6, 24–28]. Previously, we showed that mice lacking *Socs3* in myeloid cells (*Socs3*^ΔLysM^) develop a severe mixed phenotype with both cEAE and btEAE. The btEAE phenotype depends on cerebellar neutrophil infiltration and activation [24–26]. These findings suggest that myeloid SOCS3 restrains region-specific CNS inflammation and neutrophil-driven pathology.

Neutrophils are increasingly recognized as important mediators of neuroinflammation across several CNS disorders, including MS, Neuromyelitis Optica Spectrum Disorders, AD, PD, and stroke [29–38]. In MS, neutrophils display hyperactivation characterized by increased degranulation, reactive oxygen species (ROS) production, and formation of neutrophil extracellular traps (NETs) [39–41]. Neutrophils are among the earliest immune cells to infiltrate the CNS, where they disrupt the blood–brain barrier (BBB), promote demyelination, and amplify inflammatory responses [42–46]. Conversely, neutrophils can exert protective roles, such as suppressing pathogenic B-cell responses [47] and promoting axon regeneration [48–51]. While single-cell RNA sequencing (scRNA-seq) has revealed neutrophil heterogeneity in MS and cEAE [45–48, 52, 53], the diversity and function of these cells in btEAE remain poorly understood, revealing a critical knowledge gap relevant to disease mechanisms.

In this study, we generated mice with neutrophil-specific *Socs3* deletion (*Socs3*^ΔLy6G^) to define the role of neutrophil-intrinsic JAK/STAT signaling in CNS autoimmunity. *Socs3*^ΔLy6G^ mice developed both severe btEAE and cEAE, with the btEAE phenotype driven by *Socs3*-deficient neutrophils. Using single-cell transcriptomics, we identified distinct neutrophil subsets, including inflammatory clusters enriched in *Socs3*^ΔLy6G^ mice during EAE. Importantly, Saa3 emerged as a candidate effector and potential therapeutic target. Together, these findings link dysregulated neutrophil JAK/STAT signaling to brain-targeted neuroinflammation and reveal a previously unappreciated mechanism of neutrophil-mediated pathology in MS.

## MATERIALS AND METHODS

### Mice.

Transgenic mice with the *Socs3* locus flanked with flox sequences (*Socs3*^fl/fl^) [54], the generous gift of Dr. Warren Alexander (Walter and Eliza Hall Institute of Medical Research; Victoria, Australia), were bred at UAB. *Socs3*^ΔLysM^ mice were generated as previously described [25]. Mice with *Socs3* deletion exclusively in neutrophils (*Socs3*^ΔLy6G^) were generated by serial breeding of *Socs3*^fl/fl^ mice with *Ly6g*Cre^Tdtomato/+^ mice (“Catchup mice”) [55], a generous gift from Professor Matthias Gunzer, Institute for Experimental Immunology and Imaging, University Hospital, University Duisburg-Essen, Essen, Germany. The highly neutrophil-specific locus for *Ly6g* was genetically modified to drive expression of both Cre-recombinase and tdTomato. *Ly6G*^+/−^ mice serve as controls. All animal experiments were reviewed and approved by the Institutional Animal Care and Use Committee (IACUC) of UAB.

### Human Plasma Samples.

Subjects with MS and healthy controls (HC) were recruited from the UAB Comprehensive MS Center. Data were collected in a double-blinded manner until all data collection was completed. These studies were conducted in compliance with the Helsinki Declaration and approved by the Institutional Review Board at UAB. All participants provided informed consent. EDTA plasma was collected in BD Vacutainer EDTA tubes and stored at − 80°C [56]. Information on the MS patient population is summarized in Supplementary Table 1.

### Experimental Autoimmune Encephalomyelitis (EAE) Induction and Assessment.

EAE was induced in *Socs3*^fl/f*l*^, *Ly6G*^+/−^, *Socs3*^ΔLysM^ and *Socs3*^ΔLy6G^ mice by s.c. injection of MOG_35–55_ emulsified in CFA (Hooke Laboratories) along with i.p. injection of 100 ng Pertussis Toxin (Hooke Laboratories) on Days 0 and 1, as previously described [25, 26, 57]. Mice experience cEAE, btEAE, and/or a mixed phenotype after EAE induction. cEAE was scored as follows: 0, no disease; 1, decreased tail tone; 2, hind limb weakness or partial paralysis; 3, complete hind limb paralysis; 4, front and hind limb paralysis; and 5, moribund state. Assessment of btEAE was as follows: 0, no disease; 1, hunched appearance, slight head tilt; 2, ataxia, scruffy coat; 3, severe head tilt, slight axial rotation, staggered walking; 4, severe axial rotation, spinning; and 5, moribund. For mixed phenotypes, cEAE and btEAE were scored separately [26]. Mice were sacrificed on days 12–14 at the peak of EAE for histology, flow cytometry and scRNA-Seq analysis. The CB and SC were isolated as previously described [26].

### Assessment of Demyelination and NET Formation.

Mice were anesthetized and intracardially perfused with PBS, followed by 4% paraformaldehyde. CB tissues were fixed with 4% PFA at 4°C overnight and then dehydrated with 30% sucrose. Cryoprotected CB tissues were embedded in an Optimal Cutting Temperature compound and cryosectioned to produce 40 μm slices in the sagittal plane from the center. Sections were stained using the Black Gold II Myelin Staining Kit (Millipore Sigma, AG105) [26, 58]. Images of stained sections were acquired with the Keyence Microscope BZ-X800. The total *arbor vitae* (white matter) area and the myelinated area (area of black-gold staining) of the CB were measured using the “Hybrid Cell Count” module provided by Keyence Microscope. “% Cerebellar Myelination Area” was defined as the myelinated area divided by the total *arbor vitae* area. For assessment of NET formation, slides were stained for citrullinated histone 3 (citH3), a biomarker of NET formation, the neutrophil marker Ly6G, and DAPI.

### Antibodies and Cytokines.

For flow cytometry experiments, antibodies (Abs) directed against murine CD11b (M1/70), CD45 (30-F11), Ly6C (HK1.4), Ly6G (1A8), CXCR2 (SA044G4), CD62L (MEL-14), CD63 (NVG-2), CD3 (17A2), CD4 (GK1.5), CD8 (53 − 6.7), CD44 (IM7), IFN-γ (XMG1.2) and IL-17A (TC11–18H10.1) were from BioLegend (San Diego, CA). The LIVE/DEAD^®^ Fixable Aqua Stain kit (L34957) was from Thermo Fisher Scientific (Waltham, MA).

### Flow Cytometry.

For surface protein detection, cells were incubated with Fc Block (2.4G2) for 15 min. and washed, followed by incubation with viability dye and the indicated Abs, as previously described [26]. CM-H2DCFDA, a general oxidative stress indicator, was used to detect total ROS production [59, 60]. For intracellular cytokine staining, cells were stimulated with PMA (25 ng/ml) and ionomycin (1 μg/ml) in the presence of GolgiStop (BD Biosciences, San Jose, CA) for 4 h and permeabilized using the BD Fixation/Permeabilization Kit (BD Biosciences, San Jose, CA), as previously described [61].

For analysis of cells from the EAE experiments, mice were sacrificed and whole-body perfusion was performed. Mononuclear cells were isolated from the CB and SC using a 30%/70% Percoll gradient. Cell phenotypes were determined based on surface and intracellular staining patterns analyzed by flow cytometry, as previously described [23, 25, 26]. All flow cytometry data were analyzed using FlowJo software (version 10.8.1; TreeStar, Ashland, OR).

### Cytokine/Chemokine Analysis.

The supernatant of CB and SC tissue homogenates was used to determine cytokine and chemokine levels as measured by the Cytokine/Chemokine Multiplex ELISA assay (Millipore, St. Louis, MO) as previously described [62]. Analyte concentrations were normalized by protein concentration.

### Sorting of Neutrophils.

Mononuclear cells were isolated from the CB and SC of *Ly6G*^+/−^ or *Socs3*^ΔLy6G^ EAE mice as previously described [24, 63]. CD45^+^CD11b^+^Ly6G^+^Ly6C^low^ neutrophils were isolated by flow cytometry.

### Quantitative RT-PCR.

500–1000 ng of RNA from sorted neutrophils was used as a template for cDNA synthesis. qRT-PCR was performed using TaqMan primers purchased from Thermo Fisher Scientific. The resulting data were analyzed using the comparative cycle threshold method to calculate relative RNA quantities [57].

### ELISA.

The murine SAA3 ELISA kit (EZMSAA3–12K; EMD Millipore, Burlington, MA determined the concentrations of SAA3 in murine plasma and supernatants from CB and SC tissue homogenates. The human SAA1 ELISA kit (DY3019–05; R & D Systems, Minneapolis, MN) detected human SAA1 in plasma samples from MS patients and HC. The Nu.Q^®^ Discovery H3.1 ELISA kit (1001-01-03; Volition, Carlsbad, CA) quantified nucleosomes in plasma samples from MS patients and HC, as well as in murine plasma and supernatants of CB and SC tissue homogenates.

### Cell Isolation and Single-cell RNA Sequencing (scRNA-Seq).

Due to the scarcity of CD45^+^CD11b^+^ cells in the CB of naïve mice (both *Ly6G*^+/−^ and *Socs3*^ΔLy6G^) and *Ly6G*^+/−^ mice with cEAE, we could not perform scRNA-Seq on cells from the CB of these mice. Mononuclear immune cells were isolated from the SC of naïve *Ly6G*^+/−^ or *Socs3*^ΔLy6G^ mice, from the SC and CB at the peak of EAE disease at day 12 for *Socs3*^ΔLy6G^ mice, or from the SC at the peak of EAE disease at day 14 for *Ly6G*^+/−^ mice following anesthetization, intracardial PBS perfusion and a 30%/70% Percoll gradient separation [26]. Sorted live CD45^+^CD11b^+^ cells from the SC of *Ly6G*^+/−^ mice with EAE (n = 3), from the SC and CB of *Socs3*^ΔLy6G^ mice with EAE (n = 3), and from the combined SC of naïve *Ly6G*^+/−^ (n = 3), and naïve *Socs3*^ΔLy6G^ (n = 4) mice were subjected to scRNA-Seq. One biological sample consisted of samples pooled from three mice with TotalSeq^™^-B Hashtag antibodies from BioLegend [64]. All sorted CD45^+^CD11b^+^ cell libraries were prepared using the 10X Genomics Chromium Single Cell 3′ Reagent Kit V3.1 and sequenced on Illumina NextSeq 500 as previously described [45, 47, 48, 65]. Raw base call files were demultiplexed into FASTQ files. Sequencing files were processed and mapped to mm10, and count matrices were extracted using the Cell Ranger Single Cell Software (v 7.1.0) [45, 66, 67].

### scRNA-Seq Analysis.

The count matrices in the h5 file format generated from Cell Ranger were imported into the Partek Flow (Partek Inc) pipeline [66, 68]. Single-cell quality control was performed by applying an inclusion filter on counts per cell (500–15000) and detected genes per cell (250–5000). Cells with greater than 10% mitochondrial gene expression were excluded to eliminate apoptotic or dying cells [69].

The dataset was also applied by setting the noise reduction filter to exclude features where the value ≤ 0 in at least 99.9% of cells. The filtered dataset was normalized and scaled with the SCTransform workflow. Principal Component Analysis (PCA) was performed on the SCTransform-scaled data. The PCA data node was chosen to perform graph-based clustering based on the Louvain algorithm, with the number of PCA set to 20. The data was visualized using 3D Uniform Manifold Approximation and Projection (UMAP) dimensional reduction with the first 20 principal components. Cell annotations for each cluster were determined using the top differentially expressed genes (DEGs) in computed biomarkers and canonical markers following the classification workflow in Partek Flow [66, 70].

CD45^+^CD11b^+^ cells from four conditions are as follows: combined SC of naïve *Ly6G*^+/−^ (356 cells) and naïve *Socs3*^ΔLy6G^ mice (795 cells); SC of *Ly6G*^+/−^ EAE mice (4,106 cells); SC of *Socs3*^ΔLy6G^ EAE mice (11,394 cells); and CB of *Socs3*^ΔLy6G^ EAE mice (10,639 cells). The neutrophil clusters were subsetted from all of the cell clusters. Neutrophils from four different conditions are as follows: combined SC of naïve *Ly6G*^+/−^ mice (114 cells) and naïve *Socs3*^ΔLy6G^ mice (263 cells); SC of *Ly6G*^+/−^ EAE mice (2,102 cells); SC of *Socs3*^ΔLy6G^ EAE mice (6,458 cells); and CB of *Socs3*^ΔLy6G^ EAE (6,914 cells). DEGs between different samples were determined by the Hurdle model on log2-normalized counts. The dot plots and violin plots were generated with sc.pl.dotplot and sc.pl.violin functions in Scanpy (1.9.1) package [71] using the annotated h5ad files exported from Partek workflow [66].

### Pathway Enrichment Analysis.

GSEA (Gene Set Enrichment Analysis): DEG analysis between individual neutrophils versus other neutrophil clusters was performed with Gene Specific Analysis (GSA) test in Partek workflow [66]. The exported DEG list was ranked by -log(P) and converted to an RNK file, which was uploaded to GSEA software (Version 4.3.2, BROAD Institute) to run GSEAPreRanked by choosing a hallmark gene sets database [72]. The pathway analysis results were plotted in terms of normalized enrichment score (NES) and false discovery rate (FDR) using the ggplot2 (Version 3.4.0) package in RStudio.

### NicheNet Analysis.

The annotated h5ad files were read into R using the anndata package. The Seurat object [73, 74] was created for NicheNet analysis. The expression data of interacting cells was extracted from the Seurat object of integrated data. Neu1, Neu2, Neu3, Neu4 and Neu5 clusters were defined as the sender cell populations and macrophages or microglia were defined as the receiver cell populations. One comparison of interest was *Socs3*^ΔLy6G^ CB versus *Ly6G*^+/−^ SC: the condition of interest was set to *Socs3*^ΔLy6G^ CB and the reference condition was set to *Ly6G*^+/−^ SC. NicheNet analysis was performed according to the published workflow utilizing published ligand-target, ligand-receptor network and weighted integrated networks [75]. The selected differentially expressed ligand or receptor was visualized in violin plots.

### Statistics.

Significant differences between the two groups were analyzed by Student’s *t*-test distribution. One-way ANOVA was used to compare differences between more than two samples, and the Mann-Whitney rank sum test was used for EAE scores. *p*-values less than 0.05 were considered statistically significant. All error bars represent the standard error of the mean (SEM). Statistical analyses were performed with GraphPad Prism 9 (GraphPad Software, La Jolla, CA).

### Data Set Availability.

ScRNA-Seq data will be available online. The single-cell data have been deposited in the GEO under the accession number GSE304332. Raw files supporting our findings are available from the corresponding authors upon reasonable request.

## RESULTS

### Mice with Targeted Deletion of Socs3 in Neutrophils Exhibit Brain-Targeted EAE.

**Figure 1:**
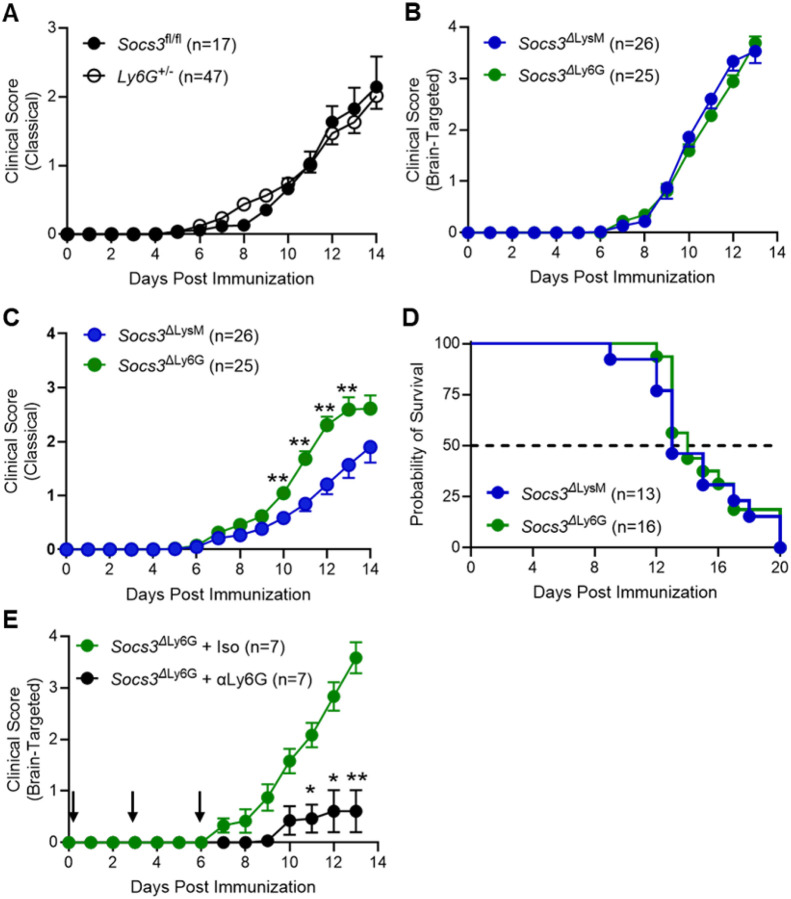
*Socs3*^Δ^Ly6G Mice Exhibit Comparable Brain-targeted EAE to *Socs3*^Δ^LysM Mice and Disease Induction Requires Neutrophils. EAE was induced in *Socs3*^fl/fl^, *Ly6G*^+/−^, *Socs3*^ΔLysM^ and *Socs3*^ΔLy6G^ mice. **(A)** Classical EAE scoring for *Socs3*^fl/fl^ and *Ly6G*^+/−^ mice. **(B)** Brain-targeted EAE scoring for *Socs3*^ΔLysM^ and *Socs3*^ΔLy6G^ mice. **(C)** Classical EAE scoring for *Socs3*^ΔLysM^ and *Socs3*^ΔLy6G^ mice. **(D)** Survival analysis for *Socs3*^ΔLysM^ and *Socs3*^ΔLy6G^ mice. **(E)** Neutrophil-specific depletion was performed in *Socs3*^ΔLy6G^ mice via neutralizing anti-Ly6G Ab (clone 1A8; 200 mg) and isotype control Ab (rat IgG2a, clone 2A3; 200 mg) administered i.p. on days 0, 3 and 6 post immunization. *p<0.05 and **p<0.01.

Our previous work established that *Socs3*^ΔLysM^ mice develop severe btEAE [26], but *Socs3* deletion in both neutrophils and macrophages precluded identifying the responsible cell type. To assess whether Socs3 deficiency in neutrophils induces brain-targeted neuroinflammation, mice with neutrophil-specific *Socs3* deletion (*Socs3*^ΔLy6G^) were generated. Effective gene deletion was confirmed by genotyping and reduced *Socs3* mRNA levels (data not shown). *Ly6G*^+/−^ mice, used as controls, showed classical EAE scores similar to *Socs3*^fl/fl^ mice (Fig. 1A) and did not develop brain-targeted EAE (btEAE), consistent with previous findings [26]. *Socs3*^ΔLy6G^ mice developed both severe btEAE and cEAE (Fig. 1B–C), with scores and survival curves closely matching those of *Socs3*^ΔLysM^ mice (Fig. 1C–D). Antibody-mediated neutrophil depletion reduced btEAE severity (Fig. 1E) but did not affect cEAE (data not shown), indicating that neutrophils are required for the brain-targeted disease phenotype. Neutrophil-specific *Socs3* deletion is sufficient to induce the mixed btEAE/cEAE phenotype previously observed in myeloid-specific knockout mice, establishing neutrophils as essential mediators of btEAE.

### Cerebellar Demyelination and Immune Cell Infiltration in Socs3^ΔLy6G^ Mice.

**Figure 2:**
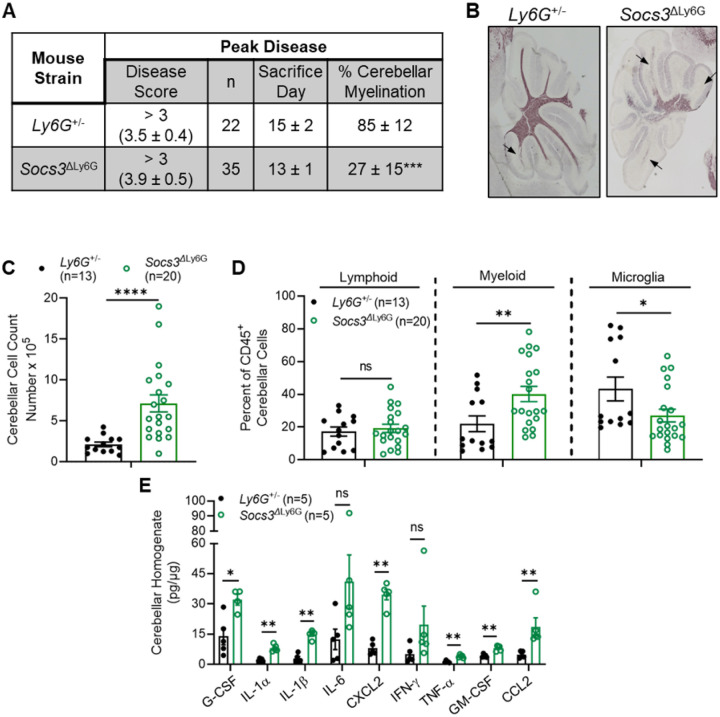
Disease Scores, Cerebellar Myelination, Cerebellar Immune Cell Infiltration and Expression of Inflammatory Cytokines and Chemokines in *Ly6G*^+/^− and *Socs3*^Δ^Ly6G Mice. EAE was induced in *Ly6G*^+/−^ and *Socs3*^ΔLy6G^ mice. **(A)** Classical EAE scores for *Ly6G*^+/−^ mice and brain-targeted EAE scores for *Socs3*^ΔLy6G^ mice, sacrifice day, and percent cerebellar myelination. **(B)** Representative cerebellar myelin staining at the peak of EAE for *Ly6G*^+/−^ and *Socs3*^ΔLy6G^ mice. Arrows indicate demyelinated regions. **(C)** CB was collected at the peak of EAE to determine immune cell populations and the inflammatory milieu in the CB. Cerebellar total cells were counted. **(D)** Lymphoid cells (CD45^+^CD11b^−^), myeloid cells (CD45^hi^CD11b^+^), and microglia (CD45^lo^CD11b^+^) percentages. **(E)** Cerebellar homogenates were collected at the peak of EAE and analyzed by Multiplex for cytokine and chemokine expression. *p<0.05, **p<0.01, ***p<0.001 and ****p<0.0001. ns: not significant.

Due to the neurological manifestations of btEAE, cerebellar pathology and immune cell infiltration were evaluated in *Socs3*^ΔLy6G^ mice. Black Gold staining demonstrated severe cerebellar demyelination at the peak of disease, with the myelinated area reduced to approximately 27% compared to 85% in controls (Fig. 2A–B). Flow cytometry confirmed increased total immune cell infiltration in the CB (Fig. 2C). There was an expansion of CD45^hi^CD11b+ myeloid cells and a reduction in CD45^lo^CD11b+ microglia (Fig. 2D). Multiplex cytokine analysis of cerebellar homogenates revealed elevated G-CSF, IL-1α, IL-1β, CXCL2, TNF-α, GM-CSF, and CCL2 (Fig. 2E). These patterns indicate a pronounced pro-inflammatory environment. *Socs3*^ΔLy6G^ mice exhibit pathology characterized by demyelination and a substantial inflammatory infiltrate, demonstrating that neutrophil-specific *Socs3* loss establishes a pathogenic immune environment in the cerebellum.

### Neutrophil Activation and NET Formation.

**Figure 3:**
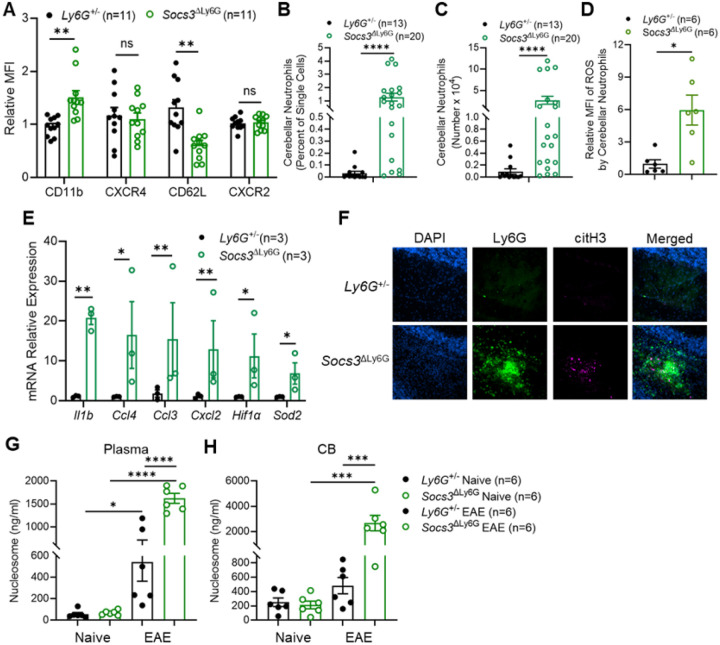
*Socs3*^Δ^Ly6G Mice Exhibit Increased Cerebellar Neutrophils and NETs. EAE was induced in *Ly6G*^+/−^ and *Socs3*^ΔLy6G^ mice. Mice were sacrificed at the peak of EAE. **(A)** CB neutrophils were subjected to surface staining. **(B)** Percent of cerebellar neutrophils (CD45^+^CD11b^+^Ly6C^low^Ly6G^+^). **(C)** Total number of cerebellar-infiltrating neutrophils. **(D)** Cerebellar neutrophils were incubated at 37°C for 30 min with CM-H2DCFDA followed by flow cytometry. **(E)** RNA was isolated from sorted cerebellar neutrophils and gene expression was analyzed by RT-PCR. **(F)** EAE was induced in *Ly6G*^+/−^ and *Socs3*^ΔLy6G^ mice. At the peak of cEAE and btEAE, respectively, the CB was fixed with 4% PFA overnight, then soaked with 30% sucrose for 3 days and embedded in OCT. NET formation was determined by staining for citrullinated histone 3 (citH3), and neutrophils were identified by Ly6G staining. Plasma **(G)** and CB **(H)** homogenate samples were collected at the peak of EAE. H3.1-nucleosome expression was quantified by ELISA. *p<0.05, **p<0.01, ***p<0.001 and ****p<0.0001. ns: not significant. MFI, mean fluorescent intensity.

The activation state and functional properties of cerebellar neutrophils were investigated in *Socs3*^ΔLy6G^ mice. Flow cytometry showed CD11b upregulation and CD62L downregulation, while CXCR4 and CXCR2 levels stayed unchanged (Fig. 3A). Both the frequency and absolute number of cerebellar neutrophils increased compared to controls, which exhibited few neutrophils in the cerebellum (Fig. 3B–C). *Socs3*^ΔLy6G^ neutrophils showed increased ROS production (Fig. 3D) and higher mRNA expression of inflammatory mediators (Fig. 3E). In inflammatory and autoimmune diseases, neutrophils release NETs, exacerbating tissue damage during sustained inflammation [76–80]. Immunofluorescence for citrullinated histone H3 demonstrated clear NET formation in cerebellar lesions (Fig. 3F), with increased nucleosome release in plasma (Fig. 3G) and cerebellar tissue (Fig. 3H) of *Socs3*^ΔLy6G^ mice. *Socs3* deficiency induces a hyperactivated, pro-inflammatory neutrophil phenotype characterized by NET formation, indicating that these cells are key effectors of CNS inflammation and demyelination.

### Spinal Cord Inflammation.

Given that *Socs3*^ΔLy6G^ mice exhibited cEAE symptoms, spinal cord (SC) pathology was also assessed. Flow cytometry indicated increased total infiltrates (Supplementary Fig. 1A), with a rise in CD11b+ myeloid cells and fewer microglia (Supplementary Fig. 1B). *Socs3*^ΔLy6G^ neutrophil frequency and numbers increased in the SC compared to controls (Supplementary Fig. 1C–D). Also, nucleosome levels were higher in both *Socs3*^ΔLy6G^ and control EAE SCs compared to naïve mice (Supplementary Fig. 1E). Neutrophil activation and NET formation were observed in both SC and CB. This suggests that *Socs3*-deficient neutrophils contribute to CNS inflammation in multiple anatomical regions.

### Th1 CD4^+^ T-cells Predominate in Socs3^ΔLy6G^ Mice.

To determine whether neutrophil-driven inflammation influences adaptive immunity, T-cell subsets were profiled in the CB and SC. *Socs3*^ΔLy6G^ mice exhibited increased total CD4+ T-cell numbers in the CB (Supplementary Fig. 2A). There was a significant elevation in IFN-γ–producing T helper 1 (Th1) cells (Supplementary Fig. 2B). A similar Th1 bias was observed in the SC (Supplementary Fig. 2C–D). Neutrophil-specific *Socs3* deletion promotes a shift of CD4+ T-cells toward the Th1 phenotype, establishing a link between neutrophil activation and adaptive immune polarization in CNS autoimmunity.

### scRNA-Seq Reveals Neutrophil Heterogeneity and Expansion of Inflammatory Subsets in Socs3^ΔLy6G^ Mice.

To study transcriptional heterogeneity in neutrophils, we performed scRNA-Seq on CNS-infiltrating cells from *Socs3*^ΔLy6G^ and control EAE mice. Cell clusters were annotated with top differentially expressed gene markers and canonical markers for neutrophils, macrophages, microglia, dendritic cells, T-cells and B-cells (Supplementary Fig. 3A). Unsupervised clustering found five neutrophil subsets (Neu1–Neu5) based on gene markers (Supplementary Fig. 3A–B).

**Figure 4:**
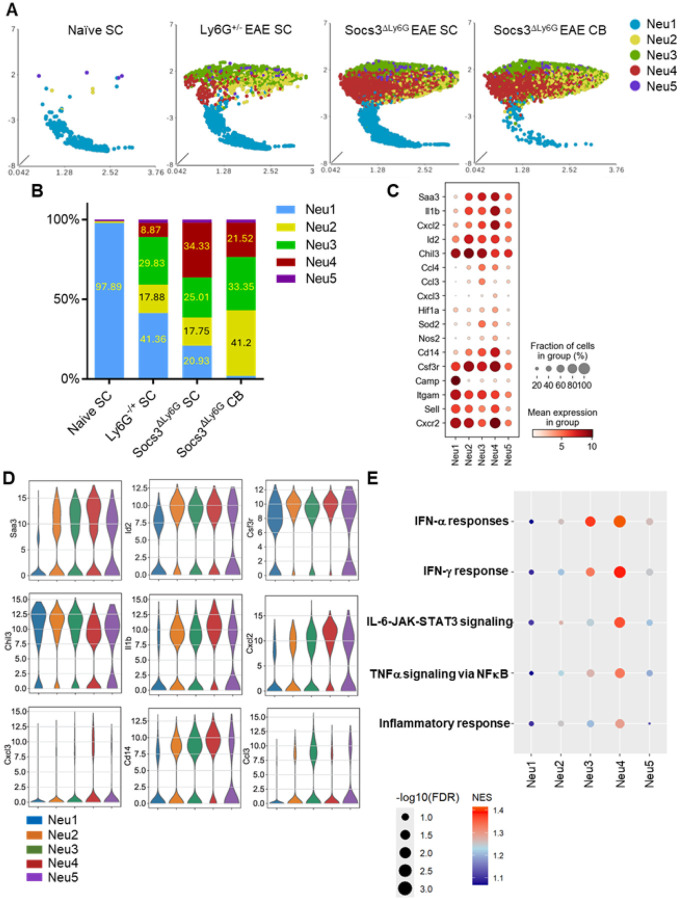
*Socs3*^Δ^Ly6G Mice Exhibit a Differential Expansion in Neutrophil Clusters Compared to *Ly6G*^+/^− Mice. EAE was induced in *Ly6G*^+/−^ and *Socs3*^ΔLy6G^ mice (n=3), and then SC and/or CB were collected at the peak of EAE (days 12–14). Live CD45^+^CD11b^+^ cells from the SC of *Ly6G*^+/−^ mice with EAE (n=3), from the SC and CB of *Socs3*^ΔLy6G^ mice with EAE (n=3), and from the combined SC of naïve *Ly6G*^+/−^ mice (n=3) and naïve *Socs3*^ΔLy6G^ mice (n=4) were subjected to scRNA-Seq. **(A)** Neutrophil clusters are shown by UMAP. **(B)** Percentage of the 5 neutrophil clusters shown in the 4 conditions. **(C)** Dot plot corresponding to neutrophil cluster genes. Dot size represents the percentage of cells in the cluster expressing the gene and dot color represents its average expression within the cluster. **(D)** Violin plots colored for differential cluster-defining genes. **(E)** Signaling pathways identified by GSEA pathway analysis in the 5 Neu clusters.

Neu1 was a quiescent group enriched in naïve SC, while Neu2–Neu4 had inflammatory signatures (Fig. 4A–D). Neu2 expressed *Saa3*, *Il1b*, *Id2*, *Chil3*, *Csf3r*, *Hist1h2bc*, and *Klhl6*. Neu3 expressed *Ccl3* and *Ccl4*. Neu4 showed an interferon and chemokine profile including *Saa3*, *Il1b*, *Cxcl2*, *Cxcl3*, *Cd14*, *Ifit1*, *Isg20*, and *Isg15* (Fig. 4C–D and Supplementary Fig. 4A–B). The top 20 marker genes from each Neu cluster are shown in Supplementary Fig. 4A. In *Socs3*^ΔLy6G^ mice, Neu2 and Neu4 clusters expanded in both CB and SC, while Neu1 was nearly absent (Fig. 4A–B). The Neu5 cluster was detected at very low percentages in all four conditions (1–2%) (Fig. 4B). Gene set enrichment showed IFNα/γ response, IL-6–JAK–STAT3, TNFα–NF-κB, and general inflammatory pathways across Neu2-5, with Neu4 most enriched for these signatures (Fig. 4E). Collectively, transcriptomic analysis reveals inflammatory neutrophil subsets expanded by *Socs3* loss, with increased STAT3- and NF-κB-driven gene programs likely driving CNS pathology.

### Transcriptomic Analysis Identifies Saa3 as One of the Highly Upregulated Genes in Socs3^ΔLy6G^ Mice.

**Figure 5:**
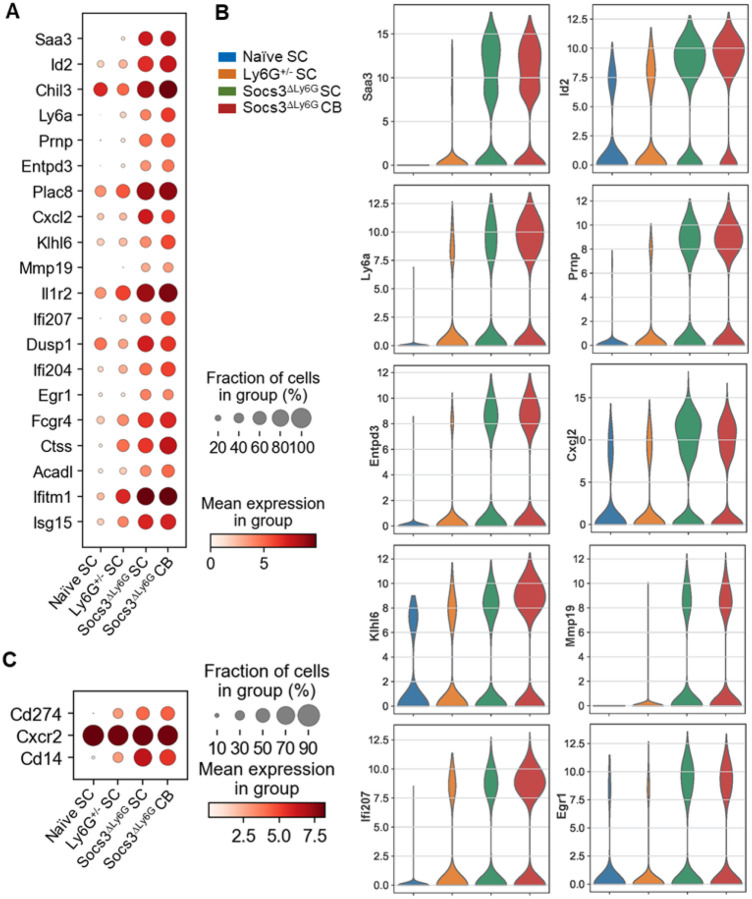
Enhanced EAE-related Gene Expression in *Socs3*^Δ^Ly6G Mice. EAE was induced in *Ly6G*^+/−^ and *Socs3*^ΔLy6G^ mice (n=3), and then SC and/or CB were collected at the peak of EAE (days 12–14). Live CD45^+^CD11b^+^ cells from the SC of *Ly6G*^+/−^ mice with EAE (n=3), from the SC and CB of *Socs3*^ΔLy6G^ mice with EAE (n=3), and from the combined SC of naïve *Ly6G*^+/−^ mice (n=3) and naïve *Socs3*^ΔLy6G^ mice (n=4) were subjected to scRNA-Seq. Neutrophils from the 4 conditions were employed for the following analyses. **(A)** Dot plot corresponding to the top 20 genes related to the 4 conditions. **(B)** Violin plots colored for differential genes in the 4 conditions. (**C)** Dot plot corresponding to genes related to neutrophil surface markers in the 4 conditions. Dot size represents the percentage of cells expressing the gene and dot color represents its average expression within the group.

DEG analysis revealed the top 20 upregulated genes in the four conditions (Fig. 5A). *Saa3*, *Id2*, *Ly6a*, *Ifi207*, *Ifi204*, *Fcgr4*, *Ctss*, *Ifitm1* and *Isg15* were increased in the SC of *Ly6G*^+/−^ mice compared to the SC of naïve mice (Fig. 5A–B). *Saa3*, *Id2*, *Chil3, Ly6a*, *Prnp*, *Entpd3*, *Cxcl2*, *Mmp19*, *Ifi207*, *Ifi204*, *Egr1*, *Acadl* and *Cd14* expression was elevated in the SC of *Socs3*^ΔLy6G^ mice compared to *Ly6G*^+/−^ mice (Fig. 5A–C). *Ly6a*, *Entpd3, Klhl6*, *Ifi207* and *Ifi204* were increased in the CB compared to the SC of *Socs3*^ΔLy6G^ mice (Fig. 5A–B). Strikingly, *Saa3* was markedly upregulated in neutrophils from the SC and CB of *Socs3*^ΔLy6G^ mice compared to *Ly6G*^+/−^ (Fig. 5A–B). *Cd14* and *Cd274* expression was elevated in neutrophils from the SC and CB of *Socs3*^ΔLy6G^ mice (Fig. 5C).

Differential gene expression analysis identified *Saa3* as one of the most highly induced genes in *Socs3*^ΔLy6G^ mice (Fig. 5A–C). *Saa3*, with *Id2*, *Chil3*, *Cxcl2*, *Mmp19*, *Ifi204*, and *Cd14*, was upregulated in both SC and CB. CB neutrophils had even higher *Saa3* expression. *Saa3* transcripts were especially high in the Neu2–Neu4 clusters (Fig. 4C and Supplementary Fig. 6). GSEA confirmed robust activation of inflammatory cytokine and interferon pathways associated with *Saa3*. *Saa3* serves as a marker of *Socs3*-deficient inflammatory neutrophils and may mediate amplification of neuroinflammation.

### Neutrophil-Macrophage/Microglia Interactions.

To investigate intercellular communication underlying CNS inflammation, ligand–receptor analysis was conducted between neutrophils and macrophage/microglia [75]. Upregulation of *Ccl4* in Neu2/Neu4 and Ccr1/Ccr5 in macrophages/microglia predicted enhanced myeloid recruitment (Supplementary Fig. 5A–C). *Saa3* also increased in neutrophils and macrophages, while *Tlr4* and *Tlr2* rose in macrophages (Supplementary Fig. 5D–E, 5G). Saa3 can activate TLR2/TLR4 to boost cytokine production [81–84]. These results suggest neutrophil-derived Saa3 activates macrophages and initiates a self-reinforcing inflammatory circuit. Saa3 induces a strong up-regulation of *Saa3* transcripts, indicating the self-amplifying potential of Saa3 [85]. *Socs3*-deficient neutrophils promote pro-inflammatory crosstalk with macrophages via Saa3–TLR2/TLR4 signaling, establishing a feed-forward loop that sustains CNS inflammation.

### Saa3 Expression is Markedly Elevated in Socs3^ΔLy6G^ Mice.

**Figure 6:**
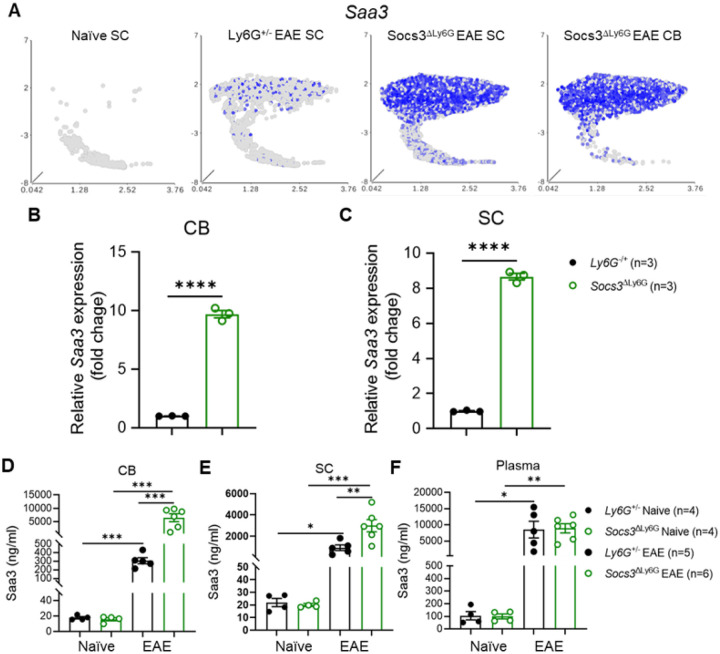
Elevated *Saa3* Expression in CB and SC of *Socs3*^Δ^Ly6G Mice with EAE Compared to *Ly6G*^+/^− Mice with EAE. EAE was induced in *Ly6G*^+/−^ and *Socs3*^ΔLy6G^ mice (n=3), and then SC and/or CB were collected at the peak of EAE (days 12–14). Live CD45^+^CD11b^+^ cells from the SC of *Ly6G*^+/−^ mice with EAE (n=3), from the SC and CB of *Socs3*^ΔLy6G^ mice with EAE (n=3), and from the combined SC of naïve *Ly6G*^+/−^ mice (n=3) and naïve *Socs3*^ΔLy6G^ mice (n=4) were subjected to scRNA-Seq. Neutrophils from the 4 conditions were employed for the following analyses. **(A)** UMAP plot of *Saa3* expression in the 4 conditions. RNA was isolated from sorted neutrophils (CD45^+^CD11b^+^Ly6G^+^Ly6C^low^) from the CB **(B)** and SC **(C)** of *Ly6G*^+/−^ and *Socs3*^ΔLy6G^ mice with EAE and *Saa3* expression was analyzed by RT-PCR. Saa3 protein expression was quantified by ELISA in tissue homogenates of CB **(D**) and SC **(E)** and in plasma **(F)** from *Ly6G*^+/−^ and *Socs3*^ΔLy6G^ naïve mice and those with EAE. *p<0.05, **p<0.01, ***p<0.001, ****p<0.0001.

Transcriptomic analysis confirmed substantial *Saa3* upregulation in the SC and CB of *Socs3*^ΔLy6G^ mice relative to controls (Fig. 6A). *Saa3* was most highly expressed in the Neu2, Neu3, Neu4, and Neu5 clusters, with the highest expression in the Neu4 cluster (Supplementary Fig. 6 and Fig. 4C–D), and was also expressed by macrophages and microglia (Supplementary Fig. 6). RT–PCR confirmed elevated *Saa3* transcripts in neutrophils isolated from both CB and SC of *Socs3*^ΔLy6G^ mice (Fig. 6B–C). At the protein level, Saa3 expression was increased in CB and SC homogenates and plasma from both *Socs3*^ΔLy6G^ and *Ly6G*^+/−^ mice with EAE compared to their corresponding naïve mice (Fig. 6D–F). The systemic Saa3 rise shows that neutrophil-specific *Socs3* deficiency induces both local and peripheral inflammation. Saa3 upregulation reflects widespread inflammatory activation following *Socs3* deletion and may serve as a biomarker of disease activity.

### SAA1 and NET Formation are Elevated in MS Patients.

**Figure 7:**
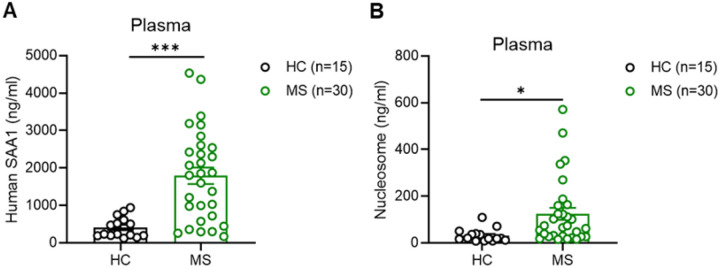
Enhanced SAA1 Expression and NET Formation in Plasma of MS Patients. **(A)** SAA1 protein expression was quantified by ELISA in plasma samples collected from MS patients and HC. **(B)** H3.1-nucleosome expression was quantified by ELISA in plasma samples collected from MS patients and HC. *p<0.05 and ***p<0.001.

To assess translational relevance, we analyzed serum from MS patients. Plasma SAA1, the human ortholog of mouse Saa3, was higher than in healthy controls (Fig. 7A). Circulating nucleosome levels, which mark NET formation, were also higher (Fig. 7B). These findings match reports of neutrophil hyperactivation in MS [39, 41, 86]. Increased SAA1 and NET markers in MS patients mirror the *Socs3*^ΔLy6G^ mouse phenotype. This links neutrophil-driven Saa3/SAA1 signaling to human CNS autoimmunity and points to therapeutic and diagnostic potential.

## DISCUSSION

Neutrophils are increasingly recognized as active regulators of neuroinflammation rather than passive bystanders. Here, we demonstrate that loss of *Socs3* in neutrophils alone (*Socs3*^ΔLy6G^ mice) drives a severe btEAE, which features cerebellar neutrophil infiltration, amplified inflammatory signaling, elevated ROS and NET formation, and extensive demyelination. Notably, depleting neutrophils completely prevented disease, establishing them as primary drivers of the brain-directed phenotype.

Single-cell transcriptomic analysis identified marked neutrophil heterogeneity within the CNS during EAE. We found five transcriptionally distinct clusters (Neu1-5) across naïve and diseased mice and observed expansion of the Neu2 and Neu4 populations in *Socs3*^ΔLy6G^ mice. The Neu4 cluster, which increased in both the SC and CB, showed enrichment for JAK/STAT and NF-κB signaling as well as interferon responses (IFNα, IFNγ). This transcriptional profile resembles interferon-stimulated neutrophil subsets previously observed in infection, autoimmunity, and chronic inflammation [87–91], pointing to a conserved inflammatory program engaged by *Socs3* deficiency.

NETs contribute to the pathogenesis of MS [79]. NETs disrupt the BBB, promote leukocyte infiltration, and exacerbate demyelination [53, 92]. Consistent with this, cerebellar neutrophils from *Socs3*^ΔLy6G^ mice released abundant NETs, accompanied by elevated expression of interferon-stimulated genes and chemokines such as *Cxcl2* (Fig. 5A–B), both of which promote NET formation [93, 94]. We also identified elevated circulating nucleosomes in the plasma of MS patients compared with healthy controls (Fig. 7B), further supporting the translational relevance of NETs as biomarkers and potential therapeutic targets.

A notable finding showed strong Saa3 activation in neutrophils from *Socs3*^ΔLy6G^ mice (Fig. 5A–B, 6A), with expression also seen in microglia and macrophages (Supplemental Fig. 6). *Saa3* levels increased in SC and CB neutrophils from *Socs3*^ΔLy6G^ mice compared with controls, indicating a role in brain targeting. Since Saa3 signals through TLR2 and TLR4, both of which are upregulated in *Socs3*^ΔLy6G^ macrophages (Supplementary Fig. 5E, 5G), our data support a feed-forward inflammatory loop between neutrophils and myeloid cells. Previous studies found that SAA-deficient mice develop delayed or milder EAE [95], highlighting the pathogenic role of this pathway. Similarly, plasma SAA1 levels were higher in MS patients than in controls (Fig. 7A), linking the murine *Saa3* axis to human disease.

Mechanistically, SOCS3 negatively regulates both NF-κB [96, 97], and JAK/STAT3 signaling [20, 22, 98], which are known drivers of *Saa3* expression [99–103]. Therefore, loss of *Socs3* removes these inhibitory controls, leading to persistent activation of these signaling pathways, upregulated Saa3 production, and amplification of inflammatory signals. SAA proteins, whose production is increased as a result, can cross and impair the intact BBB. Thus, the *Socs3*-deficient state promotes a mechanistic cascade from enhanced signaling and Saa3 production to disruption of the BBB and CNS pathology in MS.

Collectively, our findings designate neutrophils as active effectors of autoimmune neuroinflammation. *Socs3* loss triggers neutrophil activation, ROS and NET release, and Saa3 upregulation, establishing a proinflammatory circuit that drives cerebellar demyelination. By resolving neutrophil heterogeneity and defining transcriptional programs in EAE, this study highlights the Saa3/SAA1 axis as both a biomarker and a potential therapeutic target for modulating CNS autoimmunity.

## Supplementary Material

Supplementary Files

This is a list of supplementary files associated with this preprint. Click to download.

- NeutrophilsSupplTableandFigures.pdf

## Abbreviations

AD: Alzheimer’s Disease
BBB: blood-brain barrier
btEAE: brain-targeted EAE
CB: cerebellum
cEAE: classical EAE
CNS: central nervous system
DEGs: differentially expressed genes
EAE: Experimental Autoimmune Encephalomyelitis
FDR: false discovery rate
GSEA: Gene Set Enrichment Analysis
HC: healthy controls
ISG: interferon-stimulated gene
JAK: Janus Kinase
MS: Multiple Sclerosis
NES: normalized enrichment score
NETs: neutrophil extracellular traps
PCA: Principal Component Analysis
PD: Parkinson’s Disease
PTX: Pertussis Toxin
SAA: Serum Amyloid A
Saa3: Serum Amyloid A3
SC: spinal cord
scRNA-Seq: single-cell RNA Sequencing
SOCS: Suppressors Of Cytokine Signaling
STAT: Signal Transducers and Activators of Transcription
UMAP: Uniform Manifold Approximation and Projection
